# Plant cathepsin B, a versatile protease

**DOI:** 10.3389/fpls.2024.1305855

**Published:** 2024-02-23

**Authors:** Marianna Coppola, Lukas Mach, Patrick Gallois

**Affiliations:** ^1^ Faculty of Biology, Medicine and Health, University of Manchester, Manchester, United Kingdom; ^2^ Department of Applied Genetics and Cell Biology, University of Natural Resources and Life Sciences, Vienna, Austria

**Keywords:** CTS-B, programmed cell death, germination, biotic stress, abiotic stress, senescence, vacuole, apoplast

## Abstract

Plant proteases are essential enzymes that play key roles during crucial phases of plant life. Some proteases are mainly involved in general protein turnover and recycle amino acids for protein synthesis. Other proteases are involved in cell signalling, cleave specific substrates and are key players during important genetically controlled molecular processes. Cathepsin B is a cysteine protease that can do both because of its exopeptidase and endopeptidase activities. Animal cathepsin B has been investigated for many years, and much is known about its mode of action and substrate preferences, but much remains to be discovered about this potent protease in plants. Cathepsin B is involved in plant development, germination, senescence, microspore embryogenesis, pathogen defence and responses to abiotic stress, including programmed cell death. This review discusses the structural features, the activity of the enzyme and the differences between the plant and animal forms. We discuss its maturation and subcellular localisation and provide a detailed overview of the involvement of cathepsin B in important plant life processes. A greater understanding of the cell signalling processes involving cathepsin B is needed for applied discoveries in plant biotechnology.

## 1 Introduction

**Figure 1:**
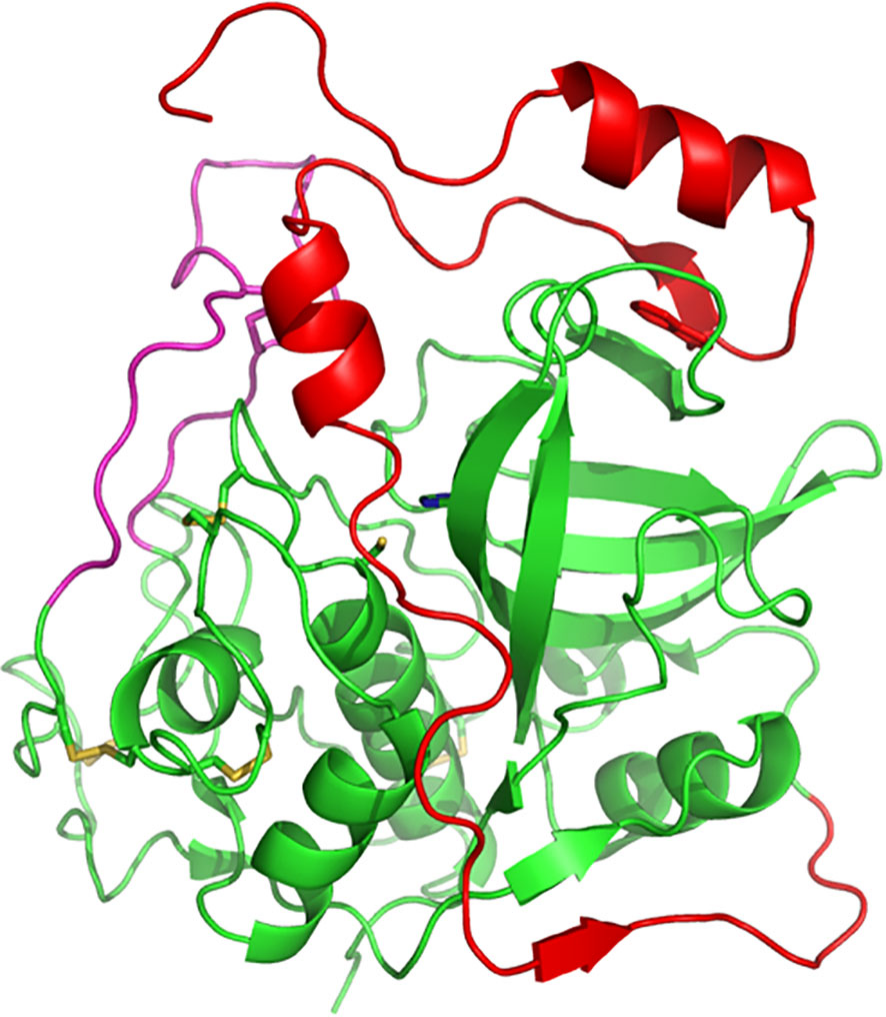
Three-dimensional structure of human procathepsin B. Structure of human procathepsin B (PDB 3PBH) showing how the propeptide (red) occupies the active-site cleft of the mature enzyme (green). The side chains of the catalytic cysteine and histidine residues are shown in stick representation. The occluding loop region is illustrated in pink. Molecular structures were prepared using PyMOL.

Over 50 years ago, Christian de Duve discovered the lysosome and, at that time, also began the study on the proteases involved in cell degradation processes within it, including cysteine cathepsins (De Duve et al., 1955). Over the following years, it was discovered that cysteine cathepsins in animal cells are involved in important biological processes and diseases such as autophagy, apoptosis, cancer, rheumatoid arthritis, atherosclerosis, heart disease and obesity (Repnik et al., 2012). In humans, there are 11 cysteine cathepsins indicated by the letters B, C, F, H, K, L, O, S, V, W, and Z (Cheng et al., 2012). In 1983, cathepsin B isolated from rat liver was first sequenced at the protein level (Takio et al., 1983), and in 1985, the first cDNA sequence of rat cathepsin B was published (San Segundo et al., 1985). Structurally, Cathepsin B is a bilobed protein with the enzyme’s active site located in the interface between the two lobes that make up the protein. Substrate cleavage is catalysed by a cysteine residue (C29) located on the left lobe that interacts with a histidine residue (H199) located on the right lobe of the enzyme (**Figure 1**). The enzymatic activity occurs in the pH range 4.0-7.4 (Musil et al., 1991; Hasnain et al., 1993).

Many research papers in the animal literature demonstrate the role of cathepsin B in the initiation, growth, angiogenesis and metastasis of murine pancreatic and mammary tumours (e.g. reviewed in (Zamyatnin et al., 2022). There is also a strong link between cathepsin B and various forms of programmed cell death (PCD). Generally, in animal models, cathepsin B silencing decreases PCD. However, a few studies also noted a survival role for cathepsin B (e.g. reviewed in (Xie et al., 2023), suggesting that cathepsin B may regulate programmed cell death negatively or positively depending on the experimental system. Overall, many reviews describe the role of cathepsin B in animal cells [e.g. (Saudenova et al., 2022)], but much less is known about its plant counterpart.

The *Arabidopsis* genome has over 800 protease genes (Van Der Hoorn, 2008) and cysteine cathepsin homologs contribute substantially to this large number. The plant cysteine proteases are divided into five clans (CA, CD, CE, CF and CP) (Richau et al., 2012). Cathepsin B (CathB) is a plant protease of the CA group, and it appears to be an important protease in key events in plant life. Here, we review both the characteristics and properties of this plant protease and its role in various processes such as plant development, germination, senescence, microspore embryogenesis, pathogen defence and responses to abiotic stress. In particular, cathepsin B is a positive regulator of stress-induced programmed cell death (PCD). We also highlight similarities and differences between plant and animal cathepsin B.

## 2 Maturation and sub-cellular localisation of plant CathB

Genes coding for cathepsin B in plants have been identified in many species, for example, *Raphanus sativus* (Tsuji et al., 2008), *Picrorhiza kurrooa* (Parkash et al., 2015), *Arabidopsis thaliana* (McLellan et al., 2009), *Nicotiana benthamiana* (Niemer et al., 2016), barley (*Hordeum vulgare*) (Gomez-Sanchez et al., 2019), and tomato (*Solanum lycopersicum* and *Solanum pimpinellifolium*) (Wen et al., 2021). In the model plant *Arabidopsis thaliana*, three loci encode CathB: *At1g02300*, AtCathB1; *At1g02305*, AtCathB2; *At4g01610*, AtCathB3 (Ge et al., 2016). Predicting the translation products of cloned transcripts derived from the *AtCathB1, AtCathB2* and *AtCathB3* genes suggested that in wild-type Col-0, AtCathB1 lacks hydrolytic activity because unexpected intron-splicing events introduce various ORF shifts that render the protease catalytically incompetent (Porodko et al., 2018). By contrast, these shifts were not detected in AtCathB2 and AtCathB3 transcripts and the expressed recombinant proteins were found to be enzymatically active (Ge et al., 2016; Porodko et al., 2018).

**Figure 2:**
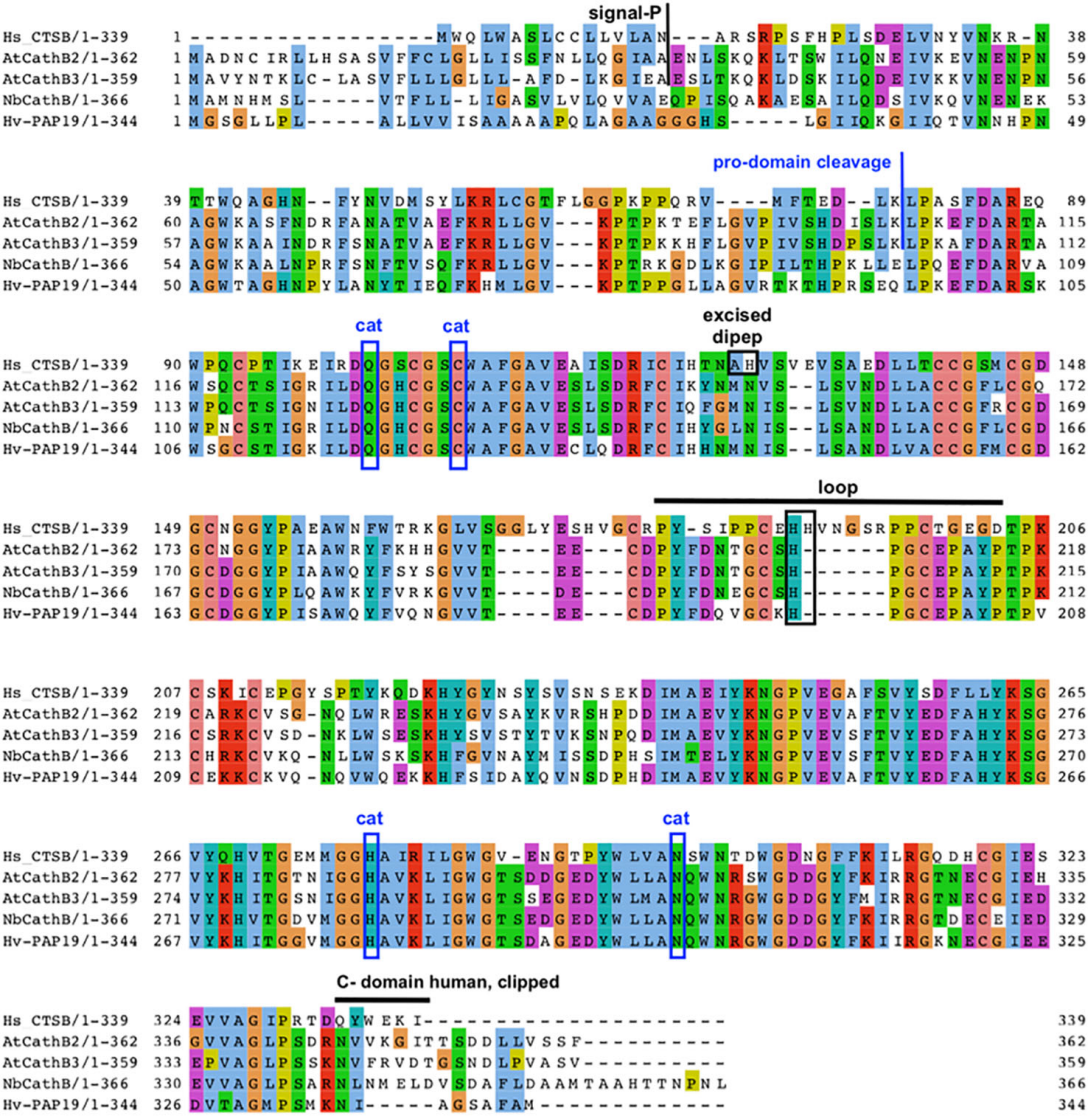
Key features of plant CathB maturation. Sequence alignment and comparison of human CathB with various plant CathB sequences: *A. thaliana, N. benthamiana* and *H. vulgare.* The signal peptide is removed during translation to form a Pro-CathB of 36 KDa. Pro-CathB is activated by the cleavage of its pro-domain and becomes a mature CathB form of 28 KDa (Porodko et al., 2018). A C-terminal domain is also removed to give a super-mature form, the precise size of which is unknown for plant CathB, as the exact cleavage site has not yet been identified (Ge et al., 2016). Finally, a di-peptide is excised in animal CathB, creating two chains. The catalytic residues are boxed (cat). The position of the occluding loop is indicated and discussed His residues are boxed. Alignment created using MUSCLE and Jalview. Clustal aa colour scheme.

Plant and animal CathB have similar structures. For example, the AtCathB2 sequence is formed by a signal peptide of 33 amino acids, a propeptide of 72 amino acids and a mature form of 257 amino acids. Evidence from analysing the expression of protein fusion constructs shows that a short C-terminal domain is clipped away during maturation, resulting in a super-mature form of around 26 kDa (**Figure 2**). The exact cleavage site for this is unknown, but C-terminal deletions of the CathB moiety in the fusion protein gave the minimum length of super-mature AtCathB2 as 240 amino acids (Ge et al., 2016).

Regarding the sub-cellular localisation of CathB in the plant cell, different results were obtained in different plant species. One of the final destinations of CathB in plants is the vacuole, which is an organelle analogous to the animal lysosome. There are two types of vacuoles in plants, the lytic vacuole (LV) and the protein storage vacuole (PSV), that perform degradation and storage functions in the plant cell. During plant development, the two different vacuoles merge to form a single vacuole that performs the functions of the LV and PSV (Carter et al., 2004). Vacuolar proteins are synthesised in the ER and then transported into the vacuole (Carter et al., 2004). Vacuolar proteases are synthesised as precursors and are expected to activate in the vacuole and then participate in the functions of the vacuole by processing or degradation of other vacuolar proteins (Gruis et al., 2004). Carter et al. (2004) characterised the vacuolar proteins in *Arabidopsis* and identified 23 proteases (cysteine proteases, aspartic proteases, serine proteases) that accumulated in this organelle, including AtCathB2 and AtCathB3. In addition, CathB has been localised in vacuoles in barley cells (Bárány et al., 2018).

In other plant species, CathB has been reported to be also present in the apoplast. In *Nicotiana benthamiana*, it has been observed that over-expressed NbCathB is located and activated in the apoplast of the cell (Gilroy et al., 2007), and in tomato, native cathepsin B is also secreted and activated in the apoplast (Gilroy et al., 2007). The location of tomato CathB in the apoplast was confirmed by Shabab et al. (2008) using activity profiling. Of note, many of the apoplastic proteases identified in tomatoes have orthologues located in the vegetative vacuole in *Arabidopsis* (Carter et al., 2004), suggesting that those proteases may take the default secretion pathway when there is downregulation of vacuolar sorting under stress for example. However, CathB secretion may also be an active and regulated process. In support of this, the localisation of cathepsin B in the apoplast of maize (*Zea mays*) is induced during fungal attacks by *Ustilago maydis* (van der Linde et al., 2012). In addition, salicylic acid (SA) treatments of maize leaves have the same effect, an observation consistent with CathB’s role in the apoplastic immunity of plants (Mueller et al., 2013).

In general, we may conclude that the different sub-cellular locations of cathepsin B in different plant species may be linked to different defence strategies using this protease.

## 3 Structure and enzymatic differences between mammalian and plant cathepsin B

Cathepsin B has both endopeptidase and peptidyldipeptidase (removal of C-terminal dipeptides) activities. Plant cathepsin B has a reduced peptidyldipeptidase activity compared to animal CathB. This difference was suggested to be linked to an occluding loop covering the enzyme’s active site, which is longer in the animal sequence than in the plant one (**Figure 2**), (Cambra et al., 2012; Niemer et al., 2016). For example, AtCathB has a six amino acid deletion in its loop area between His207 and Pro208 compared to HsCathB (**Figure 2**). The reduced exopeptidase activity in plant cathepsin B has been confirmed experimentally in daikon radish (*Raphanus sativus*) (Tsuji et al., 2008), *N. benthamiana* (Niemer et al., 2016) and *Arabidopsis* (Porodko et al., 2018).

**Figure 3:**
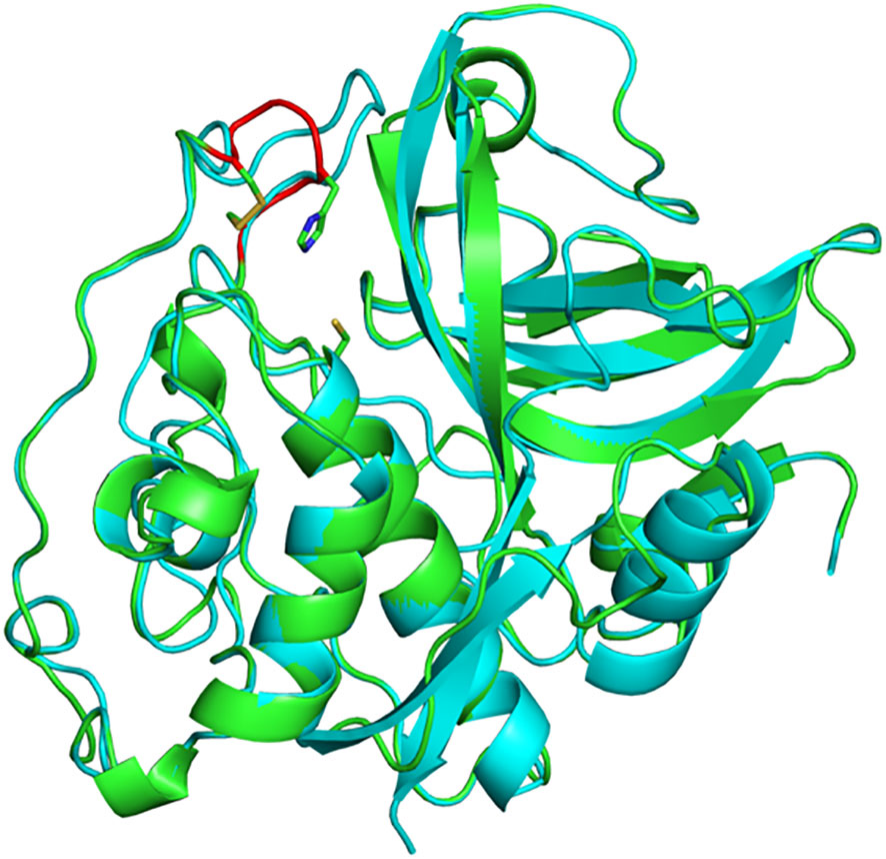
Three-dimensional structure of *A. thaliana* CathB2. Superimposition of a structural model of AtCathB2 (green) and the crystal structure of rat cathepsin B (cyan; PDB 1THE). The occluding loop of AtCathB2 is highlighted in red. The catalytic cysteine residue (Cys134) is shown in stick representation as is His207 in the occluding loop. Molecular structures were prepared using PyMOL.

In mammals, the exopeptidase activity of cathepsin B depends on two histidine residues placed in the occluding loop (**Figure 2**) (Musil et al., 1991; Krupa et al., 2002). The shorter occluding loop of plant CathB has only one histidine. Changing this histidine to alanine (His207→Ala) in AtCathB2, resulted in a marked reduction in the hydrolysis of exopeptidase and endopeptidase substrates compared to the wild-type enzyme (Porodko et al., 2018). This finding indicates that His207 is not solely required for the exopeptidase activity of the enzyme. Notably, the distance between His207 and the key amino acids Cys134 and His207 of AtCathB2 is similar to the distance between His110 and Cys29, His199 in rat cathepsin B (**Figure 3**) (Porodko et al., 2018).

It is of note that the amino acid preference at the P2 position of the substrate is different between plant and animal CathB (Paireder et al., 2017; Porodko et al., 2018). This may be due to the shorter occluding loop, which does not allow the plant enzyme to accommodate larger amino acids such as tryptophan, tyrosine and phenylalanine in the S2 subsite (Portaro et al., 2000; Krupa et al., 2002). In addition, HsCathB has a glutamic acid in its S2 subsite, which gives the enzyme a higher affinity for substrates that have an arginine residue in P2 (Hasnain et al., 1993). AtCathB3 also has a glutamic acid (Glu333) at this position, whereas AtCathB2 has a glycine (Gly336). Substituting glycine for glutamic acid (Gly336→Glu) in AtCathB2 improved its cleavage activity towards Z-Arg-Arg-MCA by increasing the affinity of the enzyme for Arg at position P2 (Porodko et al., 2018). This is consistent with the idea that the P2 position plays an important role in the substrate preferences of papain-like cysteine proteases (Richau et al., 2012). Otherwise, Porodko et al. (2018) observed that AtCathB2 showed a substantially weaker enzymatic activity than AtCathB3 on the synthetic substrates Z-His-Glu-Lys-MCA and Z-Gly-Pro-Arg-MCA.

Different cleavage preferences between CathB orthologs or paralogs can have practical implications. For example, while the human enzyme fails to cleave the monoclonal antibody (mAb) 2G12, NbCathB cleaves 2G12 (Niemer et al., 2016), showing that NbCathB is more efficient at degrading monoclonal antibodies than the human enzyme. This suggests that NbCathB is an excellent target for mutations in order to reduce unwanted proteolysis of recombinant mAbs when using the *N. benthamiana* expression platform (Niemer et al., 2016).

In conclusion, plant cathepsin B is structurally and functionally different from its mammalian counterpart, leading to the expectation that there should be plant-specific protein substrates to discover.

## 4 Inhibitors of plant cathepsin B

The differences in enzymatic activity between plant and animal cathepsin B affect the potency of inhibitors. Notably, plant CathB is far less susceptible to CA-074 inhibition than its human counterpart (Niemer et al., 2016). CA-074 needs to be used at 1 mM to obtain 100% inhibition in plants (Ge et al., 2016). Poor inhibition of plant cathepsin B by CA-074 is in agreement with its reduced exopeptidase activity, as this inhibitor was designed to exploit the peptidyldipeptidase activity that is very pronounced in the case of the human enzyme. Conversely, the inhibitory propeptide of barley cathepsin B, HvPap-19, was unable to inhibit the activity of bovine cathepsin B (Cambra et al., 2012). Both Z-FA-FMK and Ac-LVK-CHO remain effective synthetic inhibitors of plant and animal CathB (Ge et al., 2016).

Interestingly, the synthetic caspase-3 inhibitor Ac-DEVD-CHO inhibits plant Cath B to 100% at 100 µM, while the caspase-1 inhibitor Ac-YVAD-CHO causes 60% inhibition at the same concentration (Ge et al., 2016). This feature is shared with animal CathB, as Z-DEVD-FMK and Ac-YVAD-FMK inhibit the protease *in vitro* and in tissue culture (Schotte et al., 1999). Biotin-DEVD-FMK is an irreversible inhibitor that is useful for labelling plant CathB, and it has been used in pull-down experiments to identify plant CathB as one of the proteases responsible for caspase-3-like enzymatic activity in plant extracts (Ge et al., 2016).

Human cystatin C is reported as a potent inhibitor of the enzymatic activity of human cathepsin B *in vitro* (Turk et al., 1995). Its close relative chicken cystatin inhibits purified radish cathepsin B but less efficiently than human cathepsin B (Tsuji et al., 2008). There are several cystatin genes annotated in plant genomes, for example, seven genes in *Arabidopsis* and 12 in rice (Martínez et al., 2005). However, as far as we know, there is no direct *in-vitro* evidence yet that CathB inhibition by cystatins is replicated in plants. There is circumstantial evidence that three of the 13 barley cystatins inhibit an RRase (Z-RR-AMC) activity, which includes, but is not specific to, CathB activity (Martinez et al., 2009). In addition, maize cystatin-9 inhibits cathepsin B among other Cys proteases in the apoplast (van der Linde et al., 2012). Further research using plant CathB and plant cystatins, including *in vitro* assays, expression and co-localisation, may provide evidence of an additional level of regulation controlling CathB activity in plant cells.

## 5 Cathepsin B and plant pathogen defence

Plants have evolved detection mechanisms for protection against invading pathogenic organisms [reviewed in (Saijo et al., 2018)]. The recognition of a pathogenic organism by a plant cell induces a variety of defence responses, including reactive oxygen species (ROS), such as hydrogen peroxide and reactive nitrogen species (RNS), such as nitric oxide (NO) [reviewed in (Turkan, 2018)]. Responses mediated by ROS and RNS include the activation of a localised PCD characteristic of plant disease resistance hypersensitive response (HR) (Delledonne et al., 2001) that is under strict genetic control, as in the case of PCD in animal cells (Maekawa et al., 2023). It is a process that requires the use of energy and occurs in metabolically active cells (He et al., 1993). Experiments with plant cathepsin B gene silencing and overexpression have shown that this enzyme plays an important role in PCD during HR to pathogens in plants.

Gilroy et al. (2007) used cathepsin B inhibitors and silencing with VIGS to provide evidence that this papain-like protease is involved in non-host disease resistance of *N. benthamiana* induced by *Erwinia amylovora* and in HR triggered by the interaction of potato *R3a* and *P. infestans Avr3a*. By contrast, cathepsin B silencing did not show any reduction in HR following the detection of *Cladosporium fulvum AVR4* by tomato *Cf-4*, suggesting that CathB does not influence PCD in some cases. The plant response to the non-host pathogen *E. amylovora* involved an increase in CathB expression and the upregulation of *Hsr203*, a marker gene for the HR. When *CathB* was silenced, a significant reduction in the expression of *Hsr203* was observed following infection with *E. amylovora*, suggesting that cathepsin B is genetically upstream of *Hsr203*. Consistent with this observation, applying various cathepsin B inhibitors (Z-FA-FMK, Ac-LVK-CHO, CA-074-Me and Z-FGNHO-Bz) reduced the HR response. Finally, for *P. infestans*, no HR was recorded when NbCathB was silenced with VIGS, confirming the involvement of cathepsin B in the response to non-host and host pathogens in tobacco. Interestingly, a NbCathB-mRFP fusion was shown to be secreted into the apoplast, making the apoplast the likely compartment where cathepsin B regulates this process (Gilroy et al., 2007).

Subsequently, McLellan et al. (2009) used two triple mutant lines (*atcathb#62 and atcathb#57*) to knock out *AtCathB1* and *AtCathB3* by T-DNA insertion and knock-down *AtCathB2* by RNAi. These triple mutant lines demonstrated that the three CathB paralogs present in *Arabidopsis* are involved in resistance to the bacterial pathogen *Pst* DC3000, but are not required for R-gene-mediated resistance to avirulent strains of this pathogen expressing *AvrB* or *AvrRps4*. However, functional *Arabidopsis CathB* genes are required for HR triggered by *AvrB* recognition.

The involvement of Cathepsin B in response to biotic stress in plants was also confirmed in barley lines where HvPap-19 (barley CathB) was knocked-down (KD) (Gomez-Sanchez et al., 2019). KD Pap19 mutants were infected with the fungal pathogen *M. oryzae* and the phytophagous parasite *T. urticae*. Following the pathogen attack, greater leaf damage could be observed in KD Pap19 than in wild-type (WT) plants due to increased parasite colonies. However, it should be noted that the higher pathogen presence in the KD Pap19 lines might also be partially due to the reduced leaf cuticle thickness of these mutant plants compared to WT (Gomez-Sanchez et al., 2019).

In conclusion, cathepsin B is required in some forms of plant defence and HR-induced PCD, but not all. This suggests that there might be different molecular pathways involving different proteases (Salguero-Linares and Coll, 2019), and more characterisation is required to fully understand the role of cathepsin B in plant PCD.

## 6 Cathepsin B and plant senescence

Plant senescence is a controlled process closely linked to the evolution of plants, which involves PCD and is used to transfer and recycle nutrients from organs at the end of their life, [reviewed in (Woo et al., 2019)]. Papain-like cysteine proteases have been reported to be involved in the senescence phase by degrading leaf proteins to supply significant amounts of nitrogen to the plant. Among these proteases, cathepsin B, L, H, F and *SAG12* are strongly expressed during this stage of the plant life cycle (Guo et al., 2004).

McLellan et al. (2009) examined the expression of *AtCathB* genes during dark-induced senescence in *Arabidopsis*, observing that the expression of the three paralogs of *CathB* was induced after two days of dark-induced senescence. Triple *atcathb* mutants (*atcathb#62/57)* showed a delayed senescence induced by darkness compared to the wild-type Col-0, although the delay is less marked than in the positive control line, an ethylene mutant (ein-2), which displays strongly retarded senescence (McLellan et al., 2009). In particular, in the CathB mutants, the accumulation of *SAG12* transcripts after dark induction treatment is significantly decreased compared to the wild-type, suggesting that CathB is genetically upstream of this gene. In contrast, Pružinská et al., (2017) found that *atcathb* triple mutants showed no difference in senescence compared to wild-type Col-0 after seven days of darkness. This difference in results may be because, in this study, the dark-induced leaves were not detached as in McLellan et al. (2009) but remained attached to the plant and wrapped individually with aluminium foil. Different senescence induction methods, developmental or dark-induced, lead to different gene expression patterns (Van Der Graaff et al., 2006) and may highlight different pathways.

The role of cathepsin B in senescence is also supported by gene expression analysis in *Picrorhiza kurrooa* (Parkash et al., 2015). *Picrorhiza kurrooa* Royle, ex Benth, is a herb of the western Himalayan region that belongs to the *Plantaginacae* family and an endangered species (Kala, 2000). It has antioxidative, hepatoprotective, antiproliferative, immunomodulatory, antibacterial and antiviral activities (Banerjee et al., 2008). *Picrorhiza cathepsin B* was cloned (*Pk-cbcp*), and its expression level was analysed during leaf senescence. *Pk-cbcp* was seen to be expressed in all stages of leaf senescence, with a 2-fold increase in the late senescence stage compared to growing leaves. The expression levels of *Pk-cbcp* during leaf senescence were investigated in relation to phytohormones. Abscisic acid and jasmonic acid increased the expression level of *Pk-cbcp*, while cytokinin reduced the expression of the gene (Parkash et al., 2015).

Although CathB is clearly involved in leaf senescence, its exact role remains to be elucidated.

## 7 Cathepsin B in germination and microspore embryogenesis

“Germination begins with water uptake by the seed (imbibition) and ends with the start of elongation by the embryonic axis, usually the radicle” (Bewley and Black, 1994). The period during seed germination and early seedling establishment is characterised by the hydrolysis of seed storage proteins (SPP) by proteases. Protein hydrolysis begins during water uptake in the seed at the tip of the radicle in the subepidermal cell layers (Iglesias-Fernández et al., 2014). Analyses performed on *Arabidopsis* seeds have shown that there is an increased expression of genes encoding for enzymes that catalyse SSP degradation in storage vacuoles during the early stages of germination (Nakabayashi et al., 2005). In cereals, proteases are expressed in the aleurone layer, where they hydrolyse SSP in the post-germination phase; (Cejudo et al., 1992; Isabel-LaMoneda et al., 2003).

The expression level of the three genes encoding for cathepsin B-like proteases in *Arabidopsis thaliana* during seed germination was investigated (Iglesias-Fernández et al., 2014). *AtCathB3* was the most highly expressed of the three genes, especially in the post-germination period (Iglesias-Fernández et al., 2014). To analyse the role of AtCathB3 during germination in detail, the authors used single knockout (KO) lines of this gene and observed that mutant plants show slower germination than wild-type (WT) plants. Conversely, an increase in *AtCathB3* expression and faster seed germination was observed in a KO line of the transcription factor *GBF1* that represses the expression of *AtCathB3*. In addition, to localise *AtCathB3* expression during germination, a GUS (uidA) reporter line using the *AtCathB3* promoter (*PAtCathB3::uidA*) was created, and GUS expression was visible during and after germination and especially during cotyledon expansion, confirming the expression of the gene when and where SSP degradation occurs.

*In vitro* embryo development can be triggered by a cold-stress treatment on isolated microspores. After the treatment, some of the responsive microspores take an embryogenic pathway, leading to *in vitro* embryo formation (Pérez-Pérez et al., 2019). The use of an antibody against barley CathB (HvPap-19) detected the active form of the protease during cold stress-induction of microspore embryogenesis in barley and suggested a localisation in small vacuoles within the cytoplasm of the cells. An increased CathB-like enzymatic activity was also measured using the non-specific substrates Z-FR-MCA and Z-RR-MCA (Bárány et al., 2018). A reduction in PCD was observed in samples treated using the caspase-3 inhibitor Ac-DEVD-CHO, suggesting the involvement of cathepsin B in the PCD process during microspore embryogenesis in barley (Bárány et al., 2018), although it should be noted that this inhibitor also inhibits the PBA1 unit of the proteasome (Cai et al., 2018).

## 8 Cathepsin B is involved in the plant response to abiotic stresses

Protease activities are closely associated with plant responses to various abiotic stresses, in fact, the genes coding for proteases are highly expressed in response to stress caused, for example, by drought, high temperatures and excessive salt (Kidrič et al., 2014; Botha et al., 2017). Cambra et al. (2012) examined the drought responses of the C1A family (papain-like proteases, family C1, clan CA) cysteine protease (CysProt) genes in barley leaves. An analysis of the expression levels of 41 C1A CysProt barley genes after 14 days of water stress showed that HvPap-1, HvPap-8, HvPap-12, and HvPap-19 (cathepsin B) were expressed and concluded that these proteases play a role in the response to water stress in barley. Subsequently, knock-down lines for HvPap-1 and HvPap-19 kept a greater leaf turgidity during drought stress than wild-type (WT) plants, and this was at least partially linked to modified leaf structures in the mutant lines (Gomez-Sanchez et al., 2019).

Studies concerning the role of cathepsin B in abiotic stress have also been carried out on tomatoes (*Solanum lycopersicum*) (Wen et al., 2021). Two paralogs of the gene encoding for CathB2, *SlCathB2-1* and *SlCathB2-2* have been identified and characterised in tomato, and were observed to be highly expressed as a result of abiotic stress caused by drought, salt, abscisic acid, salicylic acid, jasmonic acid and especially as a result of stress caused by high temperatures (Wen et al., 2021). The experiments were conducted on the cultivated tomato line ‘LA1698′ (*Solanum lycopersicum*) and on the wild tomato line ‘LA2093′ (*Solanum pimpinellifolium*), in 6 different genotypes that are thermotolerant or sensitive to high temperatures. Stress was induced by two different temperatures of 33 and 40°C. Transcriptomic analysis showed that the two genes *SlCathB2-1* and *SlCathB2-2* are highly expressed during high-temperature stress, especially in thermosensitive genotypes, suggesting an important role for these genes in response to abiotic stress in cultivated and wild tomatoes (Wen et al., 2021).

## 9 Cathepsin B and programmed cell death

Programmed cell death (PCD) is a genetically controlled biological process that self-destructs unwanted or diseased cells. Plants use PCD for various purposes, such as the elimination of specific cells that allow the formation of fundamental plant structures, for example xylem and endosperm during plant development (dPCD), reviewed in Daneva et al. (2016), during the suppression of the suspensor in embryogenesis (Blanvillain et al., 2011), or during the formation of leaf perforations in some species (Gunawardena et al., 2004). Plants also activate the PCD pathway as a method of defence against pathogens (pPCD), which can lead to a hypersensitive response (HR) (Balakireva and Zamyatnin, 2018) where PCD is thought to block the entry of the pathogen (Greenberg and Yao, 2004). PCD in plants is also involved in the mechanisms of response to abiotic stress (ePCD), such as exposure to toxic substances, UV light or thermal shock (Danon and Gallois, 1998). In mammals, the molecular process of apoptosis activates a sequential cascade of cysteine proteases called caspases (Shalini et al., 2015). However, there are no caspases in plants, and other proteases are used in the process; (Bonneau et al., 2008; Salguero-Linares and Coll, 2019). Surprisingly, in many instances, caspase inhibitors were able to reduce or abolish plant PCD, indicating that plant proteases sensitive to these inhibitors must be involved in the process (Rotari et al., 2005). In particular, cathepsin B shows caspase-3-like enzymatic activity and is inhibited by caspase-3 inhibitors (Ge et al., 2016). Therefore, cathepsin B has been suggested to be a key protease in PCD, and this seems to be the case for stress-induced PCD. There is evidence of this in pPCD in *Nicotiana benthamiana* (Gilroy et al., 2007) and during microspore embryogenesis in barley (Bárány et al., 2018); see above. The best evidence linking cathepsin B and PCD is in ER-stress-induced PCD.

Endoplasmic *reticulum* (ER) stress occurs in plants exposed to adverse environmental conditions and is activated by misfolded proteins that accumulate within the ER; this stress triggers the unfolded proteins response (UPR), leading to the expression of proteins such as BIP and PDI to restore folding of the ER proteins, reviewed in (Howell, 2013; Howell, 2021). Under extreme or chronic conditions, unresolved ER stress can lead to PCD, referred to as ERCID (Cai et al., 2014).

In *Arabidopsis*, *AtCathB1, AtCathB2*, and *AtCathB3* have increased expression during ERCID and cathepsin B enzymatic activity is increased (Cai et al., 2018). In protoplasts of a cathepsin B triple knock-down mutant in *Arabidopsis* (*atcathb#62)*, ERCID is abolished when ER-stress is induced experimentally by the chemical tunicamycin (Ge et al, 2016). When tunicamycin is injected into leaves, ERCID is strongly reduced in *atcathb#62* (Cai et al., 2018). Concerning the process by which cathepsin B affects ERCID, there was no evidence of an interaction between CathB and VPE, another protease involved in PCD (Cai et al., 2018). VPE did not influence CathB expression, and in the VPE quadruple null mutant, cathepsin B activity remained unchanged. Conversely, in the triple mutant *atcathb#62* for cathepsin B, the activity of VPE was not affected (Cai et al., 2018).

Despite many fundamental ERCID-related steps having been studied, such as the involvement of reactive oxygen species (ROS), nuclear DNA degradation (Yang et al., 2014), and expression of proteases such as cathepsin B in the PCD process in plants (Ge et al., 2016) research on the molecular mechanisms that regulate the process is still in its nascent stages.

## 10 Conclusion

At the enzymatic level, cathepsin B has both an exopeptidase and an endopeptidase activity, representing two very different modes of interaction with a protein substrate. This suggests that cathepsin B can participate in both generic degradation of proteins and specific cleavage events. This is true for both the animal and the plant homologues. N-terminomics of rat cathepsin B has shown that the protease can degrade whole protein substrates as an endopeptidase and deactivate specific protein substrates by cleavage in the cell matrix as an endopeptidase (Prudova et al., 2016). In addition, animal cathepsin B is known to cleave specific substrates to regulate specific processes. For example, the cleavage of the apoptosis regulator BID by cathepsin B in the cytosol can trigger apoptosis (Cirman et al., 2004). We can speculate that because plant cathepsin B has a reduced exopeptidase activity compared to its animal counterpart, the specific cleavage of protein targets by the endopeptidase activity may play an even greater role in cathepsin B regulation of plant processes. Unfortunately, there is currently no known cathepsin substrate in plants, and it is a knowledge gap that our lab is addressing. Only by identifying cathepsin B substrates in plants will we be able to understand the functions that the protease carries out by activating or deactivating substrates.

At the subcellular level, cathepsin B can be located in the vacuole, the apoplast or the cytosol if released from the vacuole. Those multiple locations illustrate the versatility of the protease, which is presumably involved in various processes in various compartments. In this context, analysing the regulation of the activation of cathepsin B as it reaches various locations will be crucial to understanding its physiological role.

Finally, cathepsin B appears involved in various processes that are not connected in an obvious manner. A few processes reported in this review are clearly linked to PCD, for example, in ERCID or HR. Others are probably not linked to PCD; for example, the role of cathepsin B in early senescence or during germination, seedling establishment and barley leaf development. This suggests that cathepsin B is not specialised in a specific process and has been selected multiple times during evolution to contribute to cellular functions. However, the lack of dramatic developmental phenotypes in the triple mutant suggests that there might be enzymatic redundancy between cathepsin B and other proteases that may cleave the same substrates. This redundancy makes it more difficult to identify the important function carried out by cathepsin B using mutant genetic backgrounds. Here again, identifying substrates for either the endopeptidase or the exopeptidase activity is crucial to unravelling the function of this protease, understanding better the processes in which it is involved, and potentially manipulating these processes to benefit agriculture and biotechnology.

## Author contributions

MC: Conceptualization, Writing – original draft. LM: Validation, Writing – review & editing. PG: Writing – review & editing, Conceptualization, Funding acquisition, Supervision.
